# Causality of unsaturated fatty acids and psoriasis a Mendelian randomization study

**DOI:** 10.3389/fnut.2024.1280962

**Published:** 2024-02-09

**Authors:** Junchen Li, Qian Shen, Chenqi Guo, Yingdong Wang, Yuxiao Ma, Yu Zhang

**Affiliations:** ^1^Graduate School, Tianjin University of Traditional Chinese Medicine, Tianjin, China; ^2^Guang’anmen Hospital, China Academy of Chinese Medical Sciences, Beijing, China; ^3^Dermatology Department, Tianjin Academy of Traditional Chinese Medicine Affiliated Hospital, Tianjin, China

**Keywords:** psoriasis, polyunsaturated fatty acids, omega-3, omega-6, monounsaturated fatty acids, Mendelian randomization

## Abstract

**Background:**

Many observational studies have identified a link between unsaturated fatty acids and psoriasis. However, they contain reverse causality and confounding factors, and there is no definite causal study between unsaturated fatty acids and psoriasis.

**Objectives:**

Analysis of causality between unsaturated fatty acids and psoriasis by Mendelian randomization.

**Methods:**

We used IEU Open GWAS Project, omega-3 PUFA and omega-6 PUFA data from 114,999 subjects, MUFA data from 13,535 subjects, and psoriasis data from 4,510 cases and 212,242 controls were included. We employed the inverse-variance weighted (IVW) method as the primary analytical approach and four additional MR methods. Moreover, we performed heterogeneity and horizontal pleiotropy assessments using Cochrane’s Q and MR-Egger intercept tests, respectively. Finally, we performed sensitivity analyses to enhance our findings’ precision and veracity.

**Results:**

IVW results showed no causal effect of omega-3 PUFA on psoriasis (*p* = 0.334; OR, 0.909; 95% CI, 0.748–1.104), omega-6 PUFA cause psoriasis (*p* = 0.046; OR, 1.174; 95% CI, 1.003–1.374), MUFA cause psoriasis (*p* = 0.032; OR, 1.218; 95% CI, 1.018–1.457), no causal effect of omega-3 PUFA in psoriasis (*p* = 0.695; OR, 0.989; 95% CI, 0.937–1.044), no causal effect of omega-6 PUFA in psoriasis (*p* = 0.643; OR, 1.013; 95% CI, 0.960–1.068), psoriasis is not causal to MUFA (*p* = 0.986; OR, 1.000; 95% CI, 0.949–1.055). Heterogeneity, horizontal pleiotropy, and sensitivity analyses showed reliable results.

**Conclusion:**

We found that circulating omega-6 PUFA and MUFA cause psoriasis, while omega-3 PUFA do not. Treatments that lower circulating omega-6 PUFA and MUFA are effective in psoriasis. After a better understanding of fatty acid intake and circulation, the population can be advised to regulate their diet.

## Introduction

Lipids have traditionally been considered cell membrane structural components and metabolic energy sources. With the deepening of research, it has been found that lipids also have regulatory functions, which can regulate various cellular processes, and are of great significance to the health and disease states of the body (1). It is now recognized that lipids play an important role in psoriasis, and patients with psoriasis often have abnormalities in lipid expression and metabolism and lipid transporters and receptors (2). Mateusz et al. examined lipid-lowering therapy’s effects on psoriasis. In most patients, statins, fibrates, glitazones, and GLP-1 analogs, together with conventional psoriasis medications, relieved symptoms. However, there are also a few cases that are ineffective or aggravating for psoriasis (3). Dietary saturated fatty acids can aggravate the severity of psoriasis, while unsaturated fatty acids can relieve psoriasis to some extent (4, 5). Compared to biological agents with side effects and high economic burdens, it is safer and more cost-effective to improve psoriasis by regulating dietary lipids (6, 7). Existing research has mostly focused on polyunsaturated fatty acids, although monounsaturated fatty acids (MUFA) are also important to health (8). Various types of fatty acids may indirectly affect inflammation and neuronal signaling through adipose tissue, microbiome, intestine and vasculature. Clinical and epidemiological studies have shown that Diets rich in omega-6 polyunsaturated fatty acids (PUFA), saturated fatty acids (SFA), and trans fatty acids increase neuroinflammation. In contrast, diets rich in MUFA, omega-3 PUFA and sphingolipids can diminish neuroinflammation. However, the underlying regulatory mechanisms are multifactorial, making it difficult to establish causality (9).

Mendelian randomization studies have been used to study the causality between fatty acids and primary liver cancer (10), sepsis (11), bipolar disorder (12), and atopic dermatitis (13). Mendelian randomization studies eliminate reverse causation and confounding effects, yielding more rigorous results than observational experiments (14). Mendelian randomization discovered that blood lipids (15, 16), body mass index (17), smoking (18), and drinking (19) promote psoriasis. Unsaturated fatty acids and psoriasis are not yet linked causally. Thus, this study used Mendelian randomization to examine the bidirectional causality between omega-3, omega-6, and MUFA and psoriasis.

## Methods

**Figure 1:**
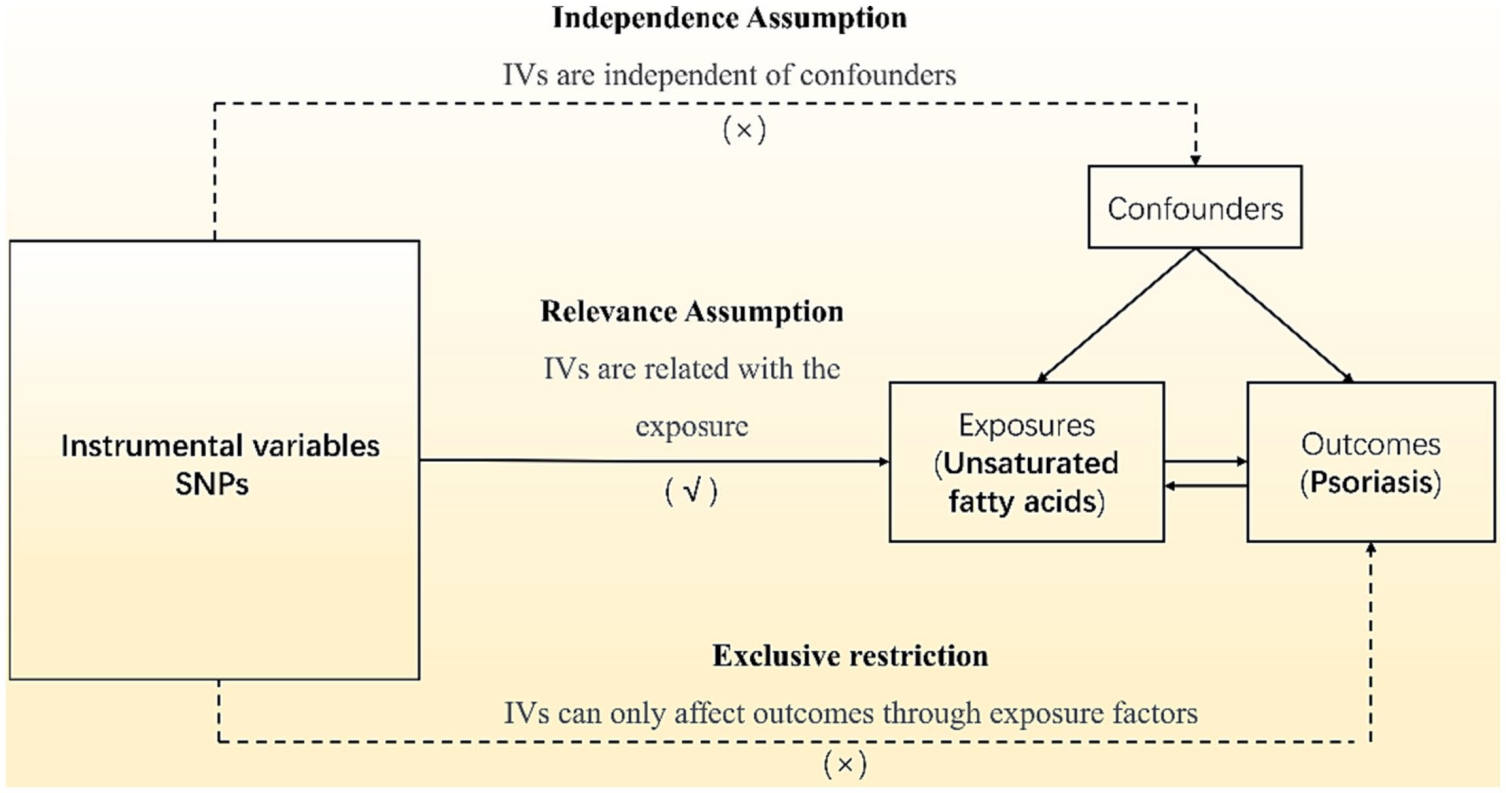
Overview of Mendelian randomization.

Our analyses employed summary data from published studies or available genome-wide association studies (GWAS) and did not require ethics committee approval. All subjects signed informed consent, and the GWAS institutional ethics review board approved each research. Omega-3 PUFA and omega-6 PUFA data use the datasets with ID met-d-Omega_3 and met-d-Omega_6 in the IEU Open GWAS Project, a European population containing males and females, including 114,999 research objects. The MUFA data uses the dataset ID met-c-916 in the IEU Open GWAS Project, a European population including males and females with 13,535 research subjects. The psoriasis data uses the dataset ID finn-b-L12_PSORIASIS in the IEU Open GWAS Project, a European population with 4,510 cases and 212,242 controls. Mendelian randomization studies used single-nucleotide polymorphisms (SNPs) as instrumental variables (IVs). Regarding the inclusion criteria for SNPs, we chose a significance threshold of *p* ≤ 5 × 10^−8^ and a minor allele frequency ≥ 3% to ensure robustness and generalizability of the findings. We excluded SNPs with reported loci overlap or linkage disequilibrium R2 < 0.001 to avoid potential confounding effects. To avoid bias from weak IVs, further screening was performed with a criterion of *F* > 10, and palindromic SNPs were eliminated to generate the final IVs. The canonical Mendelian randomization analysis followed the Strengthening the Reporting of Observational Studies in Epidemiology using Mendelian Randomization (STROBE-MR) guidelines (20). The analysis process adhered to three assumptions of Mendelian randomization studies are shown in Figure 1: (1) the correlation assumption, where the SNP is strongly associated with the exposure; (2) the exclusion assumption, where the SNP is unrelated to the outcome; and (3) the independence assumption, where the SNP is unrelated to confounding factors. Various methods, including inverse variance weighting (IVW), weighted median (WM), MR-Egger, weighted model, and simple model, were used to estimate the causal relationship between the exposure and outcome. Heterogeneity was tested using Cochran’s Q, and pleiotropy was examined through MR-Egger regression of intercept values. Additionally, PhenoScanner^1^ was utilized to detect links between genes and other diseases, aiding in the identification and exclusion of gene pleiotropy. All analyses were performed using R 4.1.2 software, incorporating packages such as “MR-PRESSO,” “Two-Sample MR,” and “MR.RAPS.”

The analysis process adhered to three assumptions of Mendelian randomization studies: (1) the correlation assumption, where the SNP is strongly associated with the exposure; (2) the exclusion assumption, where the SNP is unrelated to the outcome; and (3) the independence assumption, where the SNP is unrelated to confounding factors.

## Results

**Figure 2:**
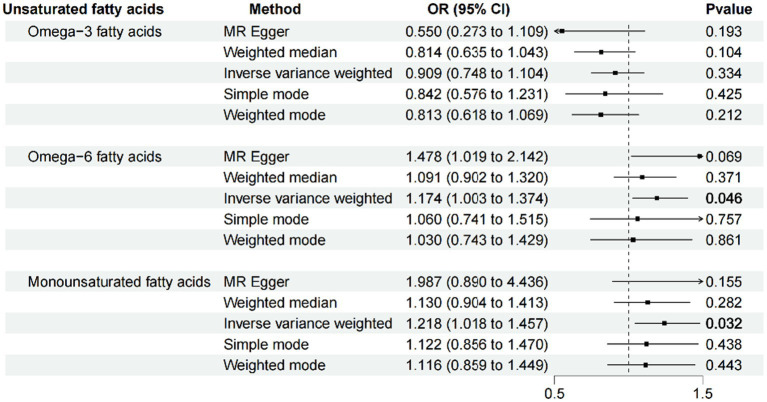
MR results (forward). IVW results showed no causal effect of omega-3 PUFA on psoriasis (*p* = 0.334; OR, 0.909; 95% CI, 0.748–1.104), omega-6 PUFA cause psoriasis (*p* = 0.046; OR, 1.174; 95% CI, 1.003–1.374), MUFA cause psoriasis (*p* = 0.032; OR, 1.218; 95% CI, 1.018–1.457), The results of MR-Egger, weighted median, simple mode, and weighted mode analyses also showed the same trend.

**Figure 3:**
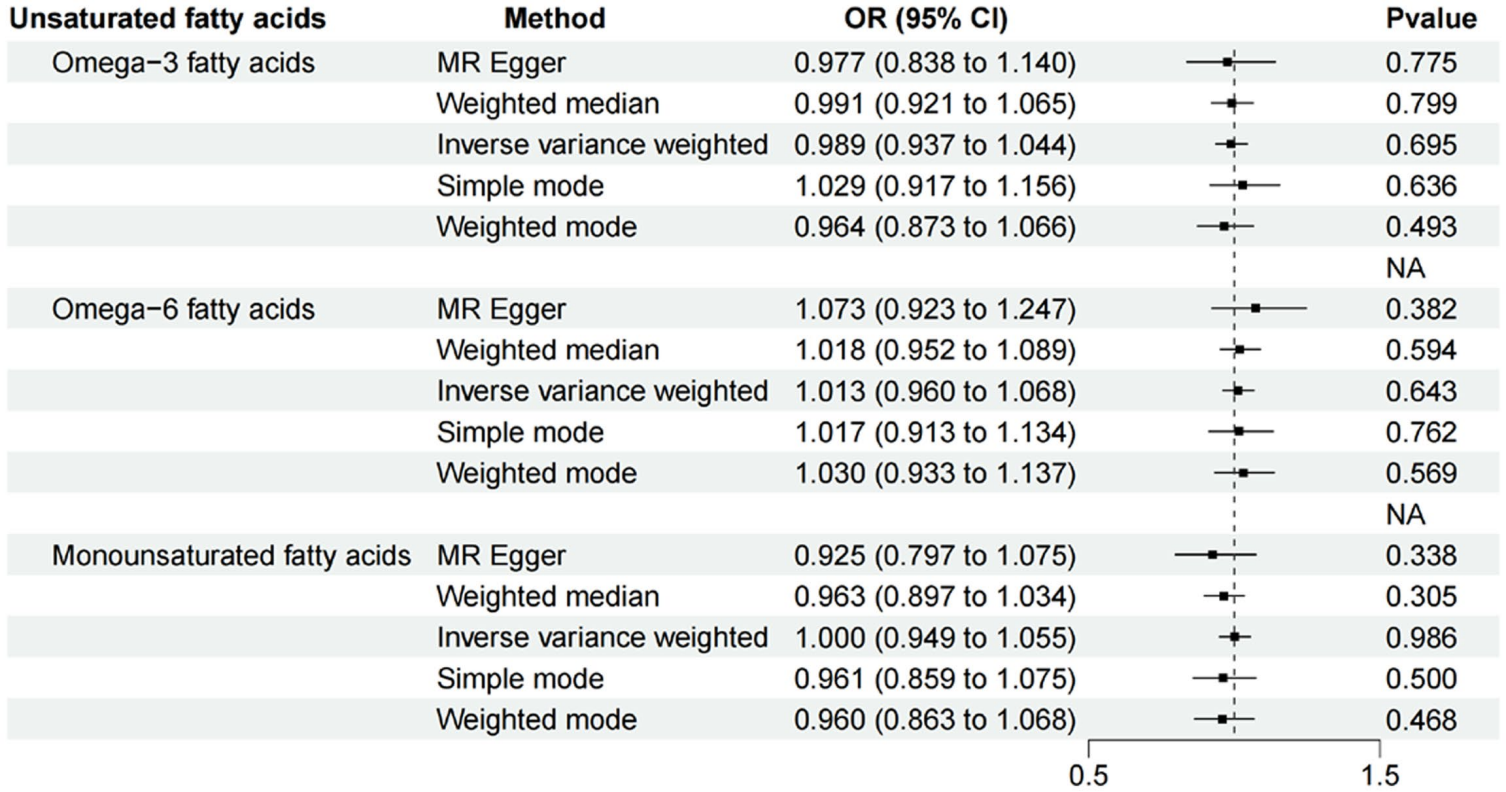
MR results (reverse). IVW results showed psoriasis not causal to omega-3 PUFA (*p* = 0.695; OR, 0.989; 95% CI, 0.937–1.044), psoriasis is not causal for omega-6 PUFA (*p* = 0.643; OR, 1.013; 95% CI, 0.960–1.068), psoriasis is not causal to MUFA (*p* = 0.986; OR, 1.000; 95% CI, 0.949–1.055). MR-Egger, weighted median, simple mode, and weighted mode analyses also show the same trend.

After ensuring the robustness, generalizability and Heterogeneity of SNPs while avoiding their potential confounding effects and pleiotropy, we selected 5, 11, and 7 SNPs as IVs for Mendelian randomization analysis between omega-3 PUFA, omega-6 PUFA, and MUFA and psoriasis, respectively. 11 SNPs were used as IVs for Mendelian randomization analysis of unsaturated fatty acids in psoriasis (Supplementary Table S1). IVW results showed no causal effect of omega-3 PUFA on psoriasis (*p* = 0.334; OR, 0.909; 95% CI, 0.748–1.104), omega-6 PUFA cause psoriasis (*p* = 0.046; OR, 1.174; 95% CI, 1.003–1.374), MUFA cause psoriasis (*p* = 0.032; OR, 1.218; 95% CI, 1.018–1.457), The results of MR-Egger, weighted median, simple mode, and weighted mode analyses also showed the same trend (Figure 2; Table 1). Global test of MRPRESSO analysis, scatter plot, Leave-one-out analysis, heterogeneity analysis and pleiotropic analysis showed that omega-6 PUFA and MUFA have causal effects on psoriasis, which is credible (Supplementary Figures S1–S4, Table S2; Table 2). IVW results showed psoriasis not causal to omega-3 PUFA (*p* = 0.695; OR, 0.989; 95% CI, 0.937–1.044), psoriasis is not causal for omega-6 PUFA (*p* = 0.643; OR, 1.013; 95% CI, 0.960–1.068), psoriasis is not causal to MUFA (*p* = 0.986; OR, 1.000; 95% CI, 0.949–1.055). MR-Egger, weighted median, simple mode, and weighted mode analyses also show the same trend (Figure 3; Table 3). The results of the Global test of MRPRESSO, qualitative and pleiotropic analysis showing no causality of psoriasis to omega-3 PUFA, omega-6 PUFA and MUFA are credible (Supplementary Table S3; Table 4).

**Table 1:** MR analysis of unsaturated fatty acids and psoriasis (forward).

	Method	nsnp	Beta	SE	*p* value	OR	95% CI
Omega-3 fatty acids	MR Egger	5	−0.598	0.358	0.193	0.550	0.273–1.109
	Weighted median	5	−0.206	0.127	0.104	0.814	0.635–1.043
	Inverse variance weighted	5	−0.096	0.099	0.334	0.909	0.748–1.104
	Simple mode	5	−0.172	0.193	0.425	0.842	0.576–1.231
	Weighted mode	5	−0.207	0.140	0.212	0.813	0.618–1.069
Omega-6 fatty acids	MR Egger	11	0.390	0.189	0.069	1.478	1.019–2.142
	Weighted median	11	0.087	0.097	0.371	1.091	0.902–1.320
	Inverse variance weighted	11	0.160	0.080	0.046	1.174	1.003–1.374
	Simple mode	11	0.058	0.182	0.757	1.060	0.741–1.515
	Weighted mode	11	0.030	0.167	0.861	1.030	0.743–1.429
Monounsaturated fatty acids	MR Egger	7	0.687	0.410	0.155	1.987	0.890–4.436
	Weighted median	7	0.123	0.114	0.282	1.130	0.904–1.413
	Inverse variance weighted	7	0.197	0.092	0.032	1.218	1.018–1.457
	Simple mode	7	0.115	0.138	0.438	1.122	0.856–1.470
	Weighted mode	7	0.110	0.133	0.443	1.116	0.859–1.449

**Table 2:** Heterogeneity and pleiotropy analyses (forward).

	Heterogeneity		MR-Egger intercept		
	Q	Q_pval	Egger_intercept	se	*p* value
Omega-3 fatty acids	3.364	0.499	0.062	0.043	0.240
Omega-6 fatty acids	13.657	0.189	−0.036	0.027	0.216
Monounsaturated fatty acids	3.890	0.692	−0.063	0.051	0.275

**Table 3:** MR analysis of psoriasis and unsaturated fatty acids (reverse).

	Method	nsnp	Beta	SE	*p* value	OR	95% CI
Omega-3 fatty acids	MR Egger	11	−0.023	0.079	0.775	0.977	0.838–1.140
	Weighted median	11	−0.009	0.037	0.799	0.991	0.921–1.065
	Inverse variance weighted	11	−0.011	0.028	0.695	0.989	0.937–1.044
	Simple mode	11	0.029	0.059	0.636	1.029	0.917–1.156
	Weighted mode	11	−0.036	0.051	0.493	0.964	0.873–1.066
Omega-6 fatty acids	MR Egger	11	0.071	0.077	0.382	1.073	0.923–1.247
	Weighted median	11	0.018	0.034	0.594	1.018	0.952–1.089
	Inverse variance weighted	11	0.013	0.027	0.643	1.013	0.960–1.068
	Simple mode	11	0.017	0.055	0.762	1.017	0.913–1.134
	Weighted mode	11	0.030	0.050	0.569	1.030	0.933–1.137
Monounsaturated fatty acids	MR Egger	11	−0.077	0.077	0.338	0.925	0.797–1.075
	Weighted median	11	−0.037	0.036	0.305	0.963	0.897–1.034
	Inverse variance weighted	11	0.000	0.027	0.986	1.000	0.949–1.055
	Simple mode	11	−0.040	0.057	0.500	0.961	0.859–1.075
	Weighted mode	11	−0.041	0.054	0.468	0.960	0.863–1.068

**Table 4:** Heterogeneity and pleiotropy analyses (reverse).

	Heterogeneity		MR-Egger intercept		
	Q	Q_pval	Egger_intercept	se	*p* value
Omega-3 fatty acids	3.809	0.956	0.003	0.016	0.871
Omega-6 fatty acids	8.584	0.572	−0.013	0.016	0.440
Monounsaturated fatty acids	9.423	0.493	0.017	0.016	0.305

## Discussion

Our findings support earlier research demonstrating omega-3 PUFAs do not cause psoriasis. Omega-3 PUFA is mainly categorized into three representative lipids: α-linoleic acid (ALA), docosahexaenoic acids (DHA), and eicosapentaenoic acid (EPA) (21). RNA sequencing data showed that the ALA metabolic pathway was significantly altered in psoriatic skin compared with normal skin (22). After dietary intervention in psoriatic mice, DHA significantly reduced circulating pro-inflammatory cytokines and bioactive lipid mediators and altered macrophage phenotypes and lipid oxidation genes. However, EPA did not exhibit this function (23). An analysis of data from the National Health and Nutrition Examination Survey (NHANES) found a potential association between daily dietary intake of eicosatetraenoic acid and a lower risk of psoriasis among U.S. adults. As the main representative lipids of omega-3 PUFA, ALA and DHA have opposite associations with psoriasis, while EPA has not been reported to have an association with psoriasis. We speculate that their effects are superimposed on each other, which ultimately leads to the lack of causality between omega-3 PUFA and psoriasis.

Contrarily, there is no such significant correlation between EPA and DHA (24). Supplementation with omega 3 PUFA alone does not significantly reduce autoimmune disease in a national, randomized, double-blind, placebo-controlled trial of older adults in the United States (25). Systematic reviews and meta-analyses also showed no association between omega-3 PUFA supplementation and improvement in psoriasis (26, 27).

Some omega-3 PUFA metabolites have anti-inflammatory properties, according to conventional wisdom. However, partially generated metabolites of omega-6 PUFA increase skin inflammation by boosting the signaling of other chemical mediators, such as cytokines and chemokines (28). However, there are still ambiguities or contradictions in the results of many studies. We cannot yet categorize omega-3 and omega-6 PUFA as good or bad for illnesses (29). Our study predicted at the genetic level that omega-6 PUFAs would cause psoriasis. This is consistent with the clinical observation that a large amount of omega-6 PUFA is detected in the blood and skin of patients with psoriasis. Omega-6 PUFA, an inflammatory mediator, is thought to cause psoriasis (30, 31).

There are few studies on MUFA, and most studies speculate that MUFA can reduce the performance of psoriasis. For example, human and animal dietary studies have shown that replacing SFA intake with MUFAs activates beneficial anti-inflammatory mechanisms (M2 macrophage polarization, adipocyte IL-10 secretion, and NLRP3 inflammasome inhibition) and reverses the harmful effects of SFAs on adipose tissue, liver tissue, and β-cells (32). Animal experiments have shown that MUFA subtype omega-9 carrying and cooperating with phosphodiesterase inhibitors can inhibit activated neutrophils, thereby reducing psoriasis-like lesions in mice (33). According to clinical observations, patients with low MUFA intake had higher PASI scores and C-reactive protein levels than those with high intake (34). However, our results show that MUFA can lead to psoriasis. Obese and non-obese psoriasis patients with or without arthritis have greater serum MUFAs than healthy people (35, 36).

Our data also suggest that omega-3 and omega-6 PUFA, and MUFA do not cause reverse psoriasis. In conclusion, omega-6 PUFAs and MUFAs genetically cause psoriasis. However, there are still some shortcomings in this study. The unsaturated fatty acid data we used for analysis were derived from serum polyunsaturated fatty acids. Although circulating fatty acids are closely related to fatty acid intake, the two cannot be completely equal. Therefore, the effect of intervening fatty acid intake on psoriasis deserves more study. This study employed European data; thus, further research is needed to determine if the results apply to other races. This study only studied at the level of omega-3 PUFA, omega-6 PUFA and MUFA. Subsequent research should be conducted with more subdivided components. Mendelian randomization is an emerging study strategy that reduces confounding and reverse causality, but the analysis is completely dependent on the gene level, and its reliability is still being tested.

## Conclusion

We found that circulating omega-6 PUFA and MUFA cause psoriasis, while omega-3 PUFA do not. Treatments that lower circulating omega-6 PUFA and MUFA are effective in psoriasis. After a better understanding of fatty acid intake and circulation, the population can be advised to regulate their diet.

## Data availability statement

The original contributions presented in the study are included in the article/Supplementary material, further inquiries can be directed to the corresponding author.

## Ethics statement

The studies involving humans were approved and employed by the summary data from published studies or available genome-wide association studies (GWAS) and did not require ethics committee approval. All subjects signed informed consent, and the GWAS institutional ethics review board approved each research. The studies were conducted in accordance with the local legislation and institutional requirements. Written informed consent for participation was not required from the participants or the participants’ legal guardians/next of kin in accordance with the national legislation and institutional requirements.

## Author contributions

JL: Conceptualization, Investigation, Methodology, Writing – original draft, Writing – review & editing. QS: Conceptualization, Investigation, Writing – original draft. CG: Investigation, Methodology, Writing – original draft. YW: Investigation, Writing – original draft. YM: Investigation, Writing – original draft. YZ: Funding acquisition, Resources, Supervision, Writing – review & editing.

## Footnotes

^1^ http://www.phenoscanner.medschl.cam.ac.uk/
