# Supplementary material for: Causality of unsaturated fatty acids and psoriasis a Mendelian randomization study
Source: Front Nutr. 2024 Feb 9;11:1280962. doi: 10.3389/fnut.2024.1280962 (PMC10884181; doi:10.3389/fnut.2024.1280962)
Supplement: Supplementary file 1 [file Table_1.DOCX]

**Supplementary Table S1. Characteristics of the genetic instrument variables for unsaturated fatty acids at the genome-wide significance level (P < 5 × 10^–8^)**

|  | **SNP** | **chr** | **pos** | **A1** | **A2** | **Beta** | **SE** | **P** | **EAF** | **BETA2** | **SE2** | **P2** | **F-statistic** |
| --- | --- | --- | --- | --- | --- | --- | --- | --- | --- | --- | --- | --- | --- |
| **Omega-3 fatty acids** | rs1077835 | 15 | 58723426 | G | A | 0.088821 | 0.014466 | 1.08E-09 | 0.249772 | 0.0086 | 0.0256 | 0.7388 | 37.69939627 |
|  | rs11604424 | 11 | 116651115 | T | C | -0.090079 | 0.014237 | 3.32E-10 | 0.756243 | 0.0094 | 0.026 | 0.717799 | 40.03226312 |
|  | rs1260326 | 2 | 27730940 | C | T | -0.096804 | 0.012677 | 3.37E-14 | 0.636884 | -0.0228 | 0.0234 | 0.3314 | 58.31142105 |
|  | rs145717049 | 19 | 19130096 | T | C | -0.191179 | 0.032739 | 6.67E-09 | 0.044158 | 0.0389 | 0.0501 | 0.4384 | 34.09962095 |
|  | rs174546 | 11 | 61569830 | T | C | -0.15381 | 0.012443 | 1.19E-34 | 0.40295 | 0.0359 | 0.0227 | 0.1134 | 152.7984477 |
|  |  |  |  |  |  |  |  |  |  |  |  |  |  |
| **Omega-6 fatty acids** | rs10402112 | 19 | 11191677 | A | T | -0.166416 | 0.021114 | 3.8E-15 | 0.099716 | -0.048 | 0.037 | 0.1937 | 62.12252845 |
|  | rs11591147 | 1 | 55505647 | T | G | -0.309146 | 0.039917 | 1.12E-14 | 0.028839 | -0.1781 | 0.0606 | 0.00331101 | 59.98069244 |
|  | rs1260326 | 2 | 27730940 | C | T | -0.07798 | 0.012722 | 9.73E-10 | 0.63681 | -0.0228 | 0.0234 | 0.3314 | 37.57125302 |
|  | rs144064722 | 4 | 73406173 | G | A | 0.23669 | 0.039503 | 2.29E-09 | 0.026411 | 0.0163 | 0.0667 | 0.8074 | 35.90043094 |
|  | rs174418 | 15 | 58687603 | C | T | -0.098225 | 0.012554 | 5.99E-15 | 0.562433 | 0.0077 | 0.0225 | 0.7334 | 61.21809716 |
|  | rs1800588 | 15 | 58723675 | T | C | 0.142532 | 0.014482 | 9.471E-23 | 0.249057 | 0.0129 | 0.0259 | 0.619001 | 96.86517604 |
|  | rs190934192 | 1 | 55334001 | A | G | -0.243968 | 0.043382 | 2.03E-08 | 0.027571 | -0.2047 | 0.1689 | 0.2256 | 31.62616933 |
|  | rs3741298 | 11 | 116657561 | T | C | -0.14319 | 0.01451 | 7.32E-23 | 0.769753 | 0.0142 | 0.0265 | 0.5918 | 97.38466021 |
|  | rs7412 | 19 | 45412079 | T | C | -0.272206 | 0.028286 | 8.059E-22 | 0.057368 | -0.1076 | 0.0495 | 0.02979 | 92.60881208 |
|  | rs76246956 | 4 | 74783906 | A | G | 0.225705 | 0.040317 | 2.35E-08 | 0.030794 | -0.0504 | 0.0541 | 0.3512 | 31.34050158 |
|  | rs79225634 | 5 | 74619639 | T | C | 0.08458 | 0.012959 | 7.52E-11 | 0.351533 | 0.005 | 0.0233 | 0.8304 | 42.5983112 |
|  |  |  |  |  |  |  |  |  |  |  |  |  |  |
| **Monounsaturated fatty acids** | rs115849089 | 8 | 19912370 | A | G | -0.121281 | 0.019965 | 1.79E-09 | 0.106937 | -0.0165 | 0.036 | 0.6462 | 36.9017455 |
|  | rs1260326 | 2 | 27730940 | C | T | -0.117539 | 0.012652 | 3.53E-20 | 0.636827 | -0.0228 | 0.0234 | 0.3314 | 86.30691962 |
|  | rs1601934 | 15 | 58671721 | A | G | -0.08766 | 0.01342 | 9.87E-11 | 0.657128 | 0.0486 | 0.2218 | 0.8266 | 42.66756915 |
|  | rs1800588 | 15 | 58723675 | T | C | 0.130012 | 0.01444 | 4.76E-19 | 0.24915 | 0.0129 | 0.0259 | 0.619001 | 81.06483291 |
|  | rs41272659 | 2 | 207634958 | A | G | -0.24469 | 0.042362 | 1.06E-08 | 0.024498 | -0.1875 | 0.0772 | 0.0151098 | 33.36410891 |
|  | rs8107974 | 19 | 19388500 | T | A | -0.149973 | 0.024893 | 2.42E-09 | 0.064753 | -0.0401 | 0.0453 | 0.3763 | 36.29707926 |
|  | rs964184 | 11 | 116648917 | C | G | -0.149938 | 0.017294 | 8.96E-18 | 0.856027 | -0.0152 | 0.0316 | 0.6312 | 75.1679174 |
