# Supplementary material for: Causality of unsaturated fatty acids and psoriasis a Mendelian randomization study
Source: Front Nutr. 2024 Feb 9;11:1280962. doi: 10.3389/fnut.2024.1280962 (PMC10884181; doi:10.3389/fnut.2024.1280962)
Supplement: Supplementary file 2 [file Table_2.DOCX]

**Supplementary Table S2. Global test of MRPRESSO analysis (forward)**

|  | **RSSobs** | **Pvalue** |
| --- | --- | --- |
| **Omega-3 fatty acids** | 20.38292338 | 0.126 |
| **Omega-6 fatty acids** | 7.863190226 | 0.552 |
| **Monounsaturated fatty acids** | 17.49971557 | 0.431 |
