# Supplementary material for: Causality of unsaturated fatty acids and psoriasis a Mendelian randomization study
Source: Front Nutr. 2024 Feb 9;11:1280962. doi: 10.3389/fnut.2024.1280962 (PMC10884181; doi:10.3389/fnut.2024.1280962)
Supplement: Supplementary file 3 [file Table_3.DOCX]

**Supplementary Table S3. Global test of MRPRESSO analysis (reverse)**

|  | **RSSobs** | **Pvalue** |
| --- | --- | --- |
| **Omega-3 fatty acids** | 4.598554701 | 0.957 |
| **Omega-6 fatty acids** | 10.1491944 | 0.622 |
| **Monounsaturated fatty acids** | 11.61946401 | 0.48 |
